# A CFA-Induced Model of Inflammatory Skin Disease in Miniature Swine

**DOI:** 10.1155/2018/6916920

**Published:** 2018-06-24

**Authors:** Nalú Navarro-Alvarez, Beatriz M. M. Gonçalves, Alec R. Andrews, David H. Sachs, Christene A. Huang

**Affiliations:** Center for Transplantation Sciences, Massachusetts General Hospital, 149 13 Street, Charlestown, MA 02129, USA

## Abstract

Similarities between porcine and human skin make the pig an ideal model for preclinical studies of cutaneous inflammation and wound healing. Complete Freund's adjuvant (CFA) has been used to induce inflammation and to study inflammatory pain in several animal models. Here, we evaluated the inflammation caused by CFA injected in different layers of skin and subcutaneous (SC) tissue in a large-animal model. The degree of inflammation was evaluated at early and late time points by visual inspection and histopathologic analysis. In addition, the side effects of CFA injections were evaluated based on clinical findings, behavioral changes, physiologic state, and (histo)pathologic lesions. Pigs were injected with CFA at the back of the neck's skin at different depths. All animals showed histologic signs of inflammation at the injection site. Animals injected SC did not show any signs of pain or distress (loss of appetite, abnormal behavior) and did not require pain medication. Inflammation was followed by measuring the area of induration beneath the skin. Animals injected into the dermis and/or epidermis demonstrated a severe inflammatory response on the skin surface with massive swelling, redness within 12hrs of CFA injection, and severe skin necrosis within a week, preventing accurate induration measurements. In contrast to animals injected SC, animals receiving intradermal and/or intraepidermal injection of CFA showed signs of distress requiring pain medication.* Conclusion*. SC injection of CFA in swine induces an inflammatory response that can be measured accurately by induration without causing unnecessary discomfort, providing a useful preclinical large-animal model of inflammatory skin disease.

## 1. Introduction

Inflammatory skin diseases are the most common problem in dermatology, affecting over 35 million Americans, who spend over $2 billion annually to treat their symptoms (https://www.aad.org/media/news-releases/burden-of-skin-disease). The process of skin inflammation is complex and is still not completely understood. The skin is the largest organ, covering an area of 1.5 – 2.0 m^2^ and accounts for around 15% of the body weight [1]. Although small mammals such as the mouse, rat, and rabbit have been used in skin inflammation studies, the relevance of these models to the human scenario is questionable due to the major anatomic and physiologic differences between their skin and that of humans. In contrast, the remarkable similarities between human and pig skin make pigs a very appropriate model for skin inflammatory conditions [2, 3]. These two species share similar patterns of hair follicles and blood vessels; both have a relatively thick epidermis, distinct rete ridges, dermal papillae, and dense elastic fibers in the dermis [4, 5]. Indeed, human and pig skin are almost indistinguishable histologically.

Animal models of tissue injury and inflammation can be subdivided into those that produce inflammation of cutaneous versus subcutaneous (SC) tissues. Complete Freund's adjuvant (CFA) has been used to induce inflammation and study inflammatory pain in several animal models [6]. These models attempt to mimic human conditions of persistent or chronic inflammatory pain.

CFA consists of mineral oil, containing a suspension of whole or pulverized heat-killed mycobacteria [7]. Its adjuvant activity results from sustained release of antigens from the oily deposit and stimulation of a local innate immune response, causing a delayed hypersensitivity reaction with an intense inflammatory reaction and hyperalgesia at the site of injection [8]. Among the reported reactions at the site of injection are local acute and chronic inflammation and granulomatous reactions, skin ulceration, local abscess, and/or tissue sloughing. Systemic reactions have also been reported, such as diffuse systemic granulomas secondary to migration of the oil emulsion, adjuvant-related arthritis, and very rarely, chronic wasting disease [9]. While these adverse effects have led to severe restrictions of its use by animal use committees, they make CFA an excellent model to study different inflammatory processes in the distinct skin layers. If the dose is limited, the feared adverse reactions caused by its injection can be minimized to the extent that it is considered acceptable in terms of animal welfare [10]. While alternatives to CFA may exist, those are not as potent as CFA which continues to be the gold standard for its reproducibility and ease of administration [11].

Inflammatory skin diseases are long-term conditions and are likely to need on-going care, often throughout a patient's life. By understanding the cellular events that are involved in skin inflammation in a preclinical animal model such as the pig, we could potentially develop therapeutic interventions to treat these diseases.

Here we have tested the feasibility of injecting CFA through different routes as a model of skin inflammation in a miniature swine large-animal model. The aim of this study was to evaluate the relation between the depth of injection and the severity of skin inflammation caused by CFA and to correlate this inflammation with the distress caused to the animals. The results of this study demonstrate that it is feasible to use CFA in pigs in a humane manner without causing alteration of the well-being of the animals if the CFA is injected well below the dermal layer and into the SC space.

## 2. Methods

### 2.1. Animals

Seven MGH miniature swine of either sex, weighing between 35 and 50 kg were used for this experiment. The animals were housed under environmentally controlled conditions and were fed pig diet and water ad libitum. All experiments were approved and performed in compliance with the Institutional Animal Care and Use Committee. Animals were housed at the Center for Transplantation Sciences in accordance with the Guide for the Care and Use of Laboratory Animals. All animal experiments were conducted with the approval of the Institutional Animal Care and Use Committee (IACUC) of the Massachusetts General Hospital.

### 2.2. Complete Freund's Adjuvant Injection in Miniature Swine

Complete Freund's adjuvant (1 mg/ml of mycobacterial components) was used. Prior to injection, animals underwent sedation with 10mg/kg of IM ketamine, followed by maintenance on isoflurane. All injections were accomplished on shaved areas. The skin was cleaned with povidone iodine scrub and 70% alcohol. Two routes of injection were tested: intraepidermal/dermal (n=2) and SC (n=5). One of the animals (pig# 3) underwent 8 different injections using varying needle sizes to test different CFA injection depths into the SC space.

Induction of the inflammatory reaction was achieved by the injection of well resuspended Complete Freud Adjuvant (CFA) without prior emulsification in 4 different areas at the nape of the neck on both right and left side. The pattern of injection was as shown in Figure 3. Injection sites were named 1 to 4. In each injection site 0.5ml of CFA was injected, for a total of 2ml per right or left area. The left side was used for weekly biopsies, whereas the right side was left intact and used only for measuring temperature and induration. Injections were performed using different needle size depending on the route to be tested ranging from 25g-27g, which are 15.8 mm and 12.7 mm, respectively, with and without the addition of a block of 7mm to achieve a more superficial injection (Figure 3(b)). All animals were injected with the same lot of CFA, with the exception of pig# 6 which was injected with two different lots of CFA to assess whether difference in induration or reaction could be related to different CFA lots used.

### 2.3. Clinical Assessment

Following CFA injection, we used the initial body weight as a reference value, to assess the loss or gain of weight over time, which was represented as a percentage of weight loss. In addition, body temperature, appetite, and behavior were parameters used for the clinical assessment in our animals. Pain and distress were assessed when all injection sites were palpated to subjectively determine whether the manipulation of the lesions caused discomfort to the pigs.

### 2.4. Macroscopic and Histopathologic Evaluation

All animals underwent skin biopsy prior to and by day 9 after CFA injection to monitor the levels of evolving inflammation. Two (3-6 mm) punch biopsies were taken from skin surrounding the area of CFA injection on the days mentioned above. Samples were stored in formalin, processed, and stained with H&E. Histopathologic characteristics of skin inflammation were blindly assessed by a pathologist. Additionally, before biopsy, pictures were taken, and redness, induration, and temperature were assessed.

## 3. Results

### 3.1. Effect of CFA Injection on Clinical Behavior

None of the CFA injected animals appeared acutely ill during the study. Animals that received SC CFA injections appeared healthy and did not show signs of pain or distress such as loss of appetite. Regardless of injection route, animals in this study gained weight in a time dependent manner with the exception of SC injected pig # 5 which lost only 0.7% of his initial weight (Table 1). The pain was evaluated by vocalization and avoidance reaction to touch of the lesions. SC injected animals did not react when touching their lesions, and did not appear to be in distress or require any pain management. However, the two pigs injected with CFA intraepidermally and intradermally (pigs# 1 and 2, respectively) appeared in pain judged by their vocalization and avoidance reactions when touching their injection sites. A fentanyl patch was administered to these animals to provide pain coverage without affecting the inflammatory response (Table 1).

### 3.2. Injection Depth of CFA Determines the Degree of Skin Damage and Inflammation

**Figure 1:**
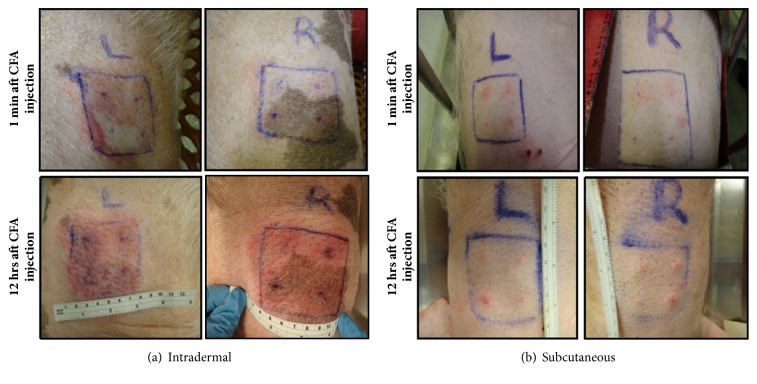
Early reaction of CFA injection after intradermal injection (a) and SC injection (b). Figure depicts both sites of injection at 1 min and 12hrs after CFA injection.

Macroscopically, inflammation as measured by redness and induration was consistently present at the sites of inoculation, but the severity was dependent on the route and depth of injection as measured by visual inspection (Figure 1) at early time points. Cutaneous inflammation appears within 12hrs after CFA injection (Figure 1). The area is demarcated by severe redness and a large ratio of induration in animals that underwent intradermal injection (Figure 1(a)); in sharp contrast, only a small pimple was visible and felt when animals were injected SC (Figure 1(b)).

**Figure 2:**
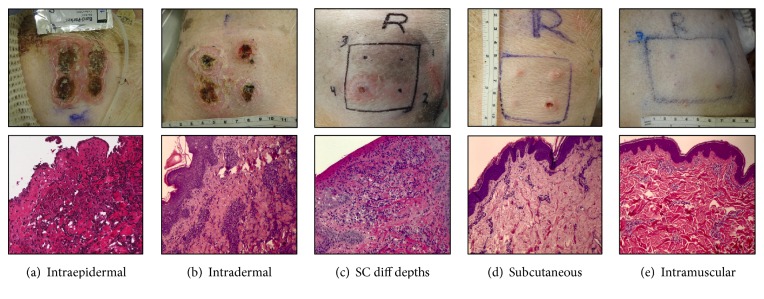
Macroscopic appearance of CFA injection into different cutaneous spaces at day 9 after injection (a) and histologic evaluation of the inflammatory infiltration (b).

Figure 2 shows that even at a later time point (day 9), the degree of inflammation and skin damage is dependent upon the depth of injection. Intraepidermal injection caused severe induration, redness, and elevation in skin temperature; macroscopically, tissue necrosis, ulceration, and scaring was easily observed at the 4 injection sites by Day 9 post CFA injection (Figure 2(a)); histologic analysis showed sloughing of the epidermal layer and massive inflammatory infiltration not only in the epidermis but in the dermal layer. Moreover, formation of small granulomas was already present as well (Figure 2(a)). Intradermal injection performed on pig #2 also resulted in tissue necrosis and denudation of the epithelial layer macroscopically and microscopically, with the presence of severe inflammatory infiltration in the epithelial and dermal layers (Figure 2(b)). When injections were performed SC, the degree of macroscopic damage depended on the depth of injection. For instance, site #4 which was the most superficial injection done at 5.7mm depth resulted in severe induration, redness, a small ulceration, and changes in temperature (Figure 2(c)). Histological evidence of inflammatory infiltrates was found in the epidermal and dermal layers. When the injection was performed SC at a depth of approximately 15.8 mm, the macroscopic appearance of the skin inflammation was less severe, and on palpation, only small indurated nodules were felt (Figure 2(d)). Histologically, the inflammatory infiltration was mainly observed in the SC layer with the presence of granulomas beginning to form at day 9 after injection; presence of small foci of infiltration was sometimes present at the dermal layer (Figure 2(d)). Finally, one animal was unintentionally injected in the muscle layer due to a difference in thickness of its SC layer. Macroscopic appearance of skin inflammation was barely visible, and on palpation, only small blebs of induration were felt. Foci of inflammatory infiltration were observed histologically in the SC tissue, with granulomatous inflammation which was more extensive involving the skeletal muscle layer. Giant cells were also observed within the granulomas, in addition to large areas of central necrosis (Figure 2(e)).

**Figure 3:**
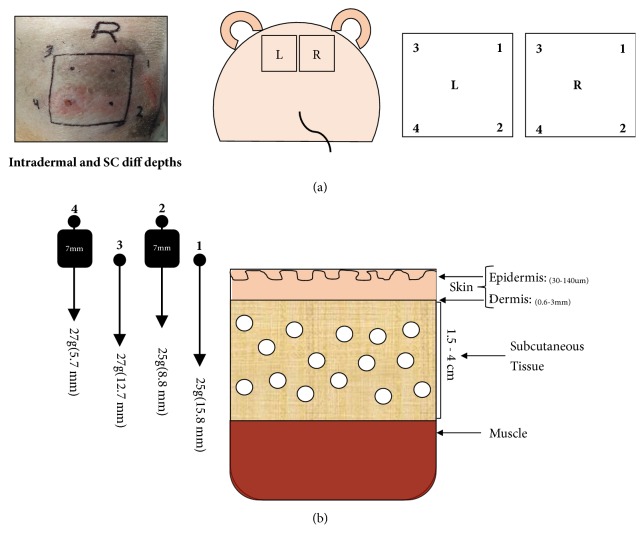
Schematic and real picture representation of the injection sites utilized for CFA administration (a). Schematic representation of the skin layers and thickness to depict the different injections depths used in this model (b).

To determine whether the degree of skin inflammation was related to the injection depth, and not individual animals reacting differently to CFA injection, 4 different SC depths were tested in the same animal in duplicate (Figure 3(a)). 2 different needle gauges were used (Figure 3(b)). Site 1 was injected with a 25g (15.8mm), which based on the length of the needle ended up in the SC tissue at 15.8mm. Site 2 was injected with the same needle, but with the addition of a 7mm block, resulting in an injection depth of 8.8mm into the SC space (15.8-7mm=8.8mm). Site 3 was injected using a 27-gauge needle with a length of 12.7mm, indicating an injection site at 12.7mm deep into the SC tissue. And lastly, injection site 4 was done in a similar manner as #3, but with the addition of a 7mm block, indicating the injection was performed at 5.7mm deep and therefore into the dermal space. This was the most superficial injection resulting in a more severe inflammatory reaction when observed macroscopically (Figures 3(a) and 3(b)).

### 3.3. Histologic Changes after Different Injection Depths

**Figure 4:**
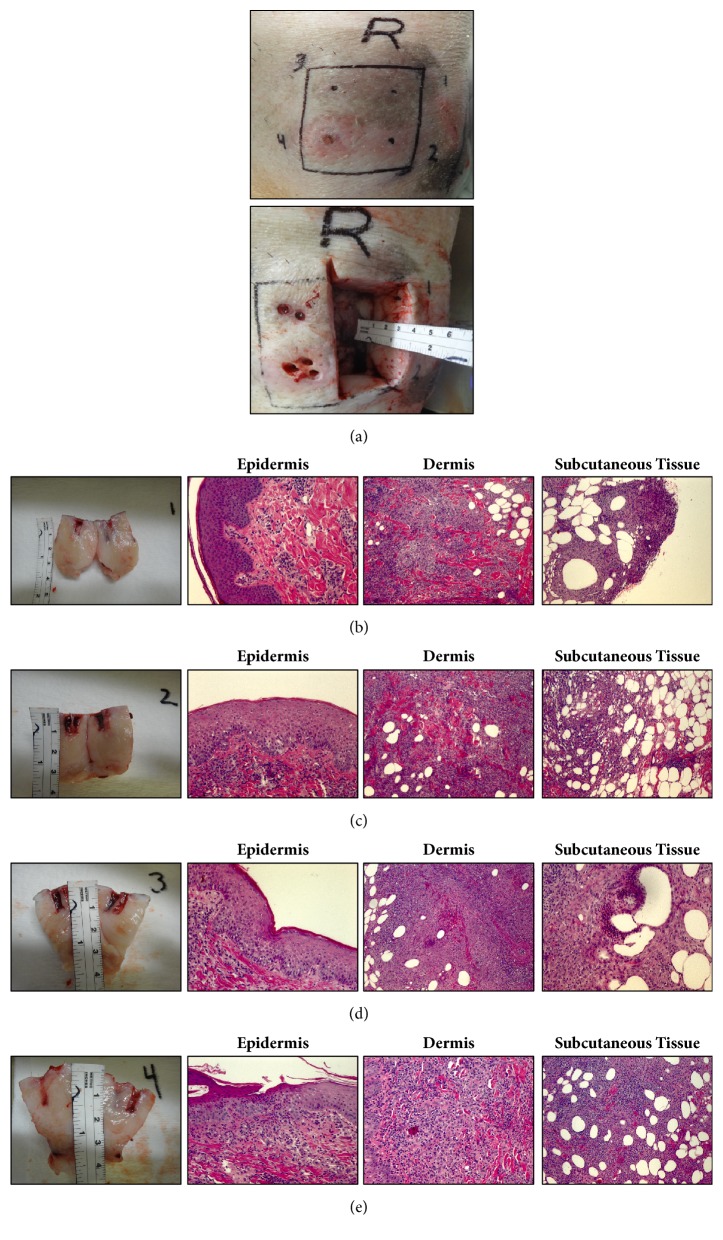
Macroscopic appearance of the skin following CFA injection into 4 different depths and picture of autopsy depicting the depth of the SC tissue in this particular animal (a). Figures (b–e), macroscopic and histologic appearance of the 4 different injection sites performed at different depths, demonstrating the inflammation in the different layers, epidermis, dermis, and SC tissue.

As illustrated in Figure 3 and confirmed by dissection shown in Figure 4(a), the skin thickness comprising all three layers ranges between 1.5 and 4 cm. CFA in injection site #1 at 15.8mm depth in pig #3 entered mainly the deep SC space where most of the inflammatory infiltration was localized. The presence of loosely organized epithelioid granulomas was also observed at this level by day 9 after injection, some of them containing central necrosis and giant cells. There was some degree of inflammatory cellular infiltration in the dermis as well (Figure 4(b)). In injection site #2 at 8.8mm depth, there was a considerable degree of cellular infiltration at the dermal layer and also in the SC tissue, where some granuloma formation was also present (Figure 4(c)). In injection site #3 at 12.7 mm depth, infiltration was mainly present in the SC space and granuloma formation occurred with some tissue necrosis. In addition, infiltration was also present in the dermis (Figure 4(d)). And finally, injection site #4 at 5.7 mm depth closest to the dermal layer presented with loss of the epidermis due to necrosis and severe perivascular inflammatory infiltrate in the superficial and deep dermis (Figure 4(e)).

### 3.4. Macroscopic Granuloma Formation after CFA Injection into SC Tissue and Muscle

**Figure 5:**
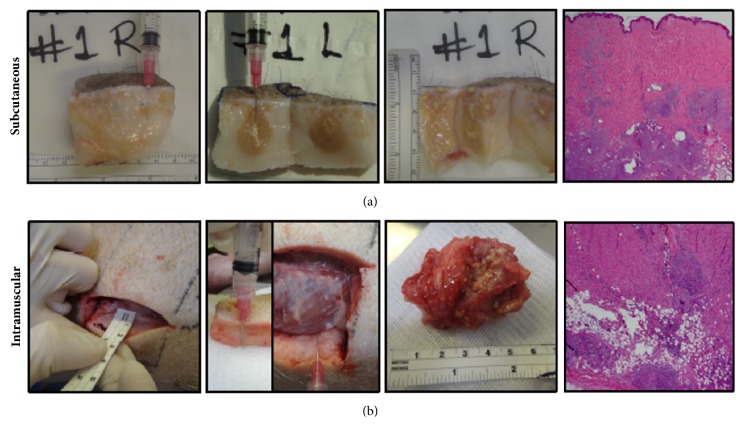
Macroscopic appearance and histologic analysis of the granulomas formed at autopsy, when CFA was injected SC (a) and (b) after intramuscular injection.

After injection of CFA using the 25g needle, which is 15.8mm long, the CFA caused massive granuloma formation into the SC space of pigs whose skin thickness ranged between 3 and 4 cm (Figure 5(a)); such massive granulomas occupied the whole length of the SC space (3-4cm) (Figure 5(a)). We confirmed the presence of granulomas in the SC tissue histologically, with involvement of the deep dermis and focally in the superficial dermis. However, when the thickness of the skin was less than 3cm, such as in the case of pig #7, the CFA was injected into the muscle layer despite using the same needle length. Muscular injection of CFA caused a massive granuloma of approximately 5 cm (Figure 5(b)). Histologically, we observed some level of inflammatory infiltration into the deep SC tissue but the majority of inflammation was in the muscle, where massive granuloma with giant cells and central necrosis was observed (Figure 5(b)).

## 4. Discussion

Unlike skin of rodents, dogs, or nonhuman primates, porcine skin is similar to human skin in terms of structure of epidermal rete ridges, hair follicle structure and density, and presence of sweat glands and subcutaneous fat [12]. For this and other reasons, the pig is considered as an excellent translational animal model in many fields of biomedical research [3, 13] including studies of inflammation [14, 15] and cutaneous graft versus host disease [16]. Complete Freund's Adjuvant is a water in oil emulsion containing killed, dried Mycobacterium which has been used as an adjuvant to enhance antigenicity and augment an immune response [8, 17, 18]. CFA has been used in several inflammatory models due to its ability to elicit an intense inflammatory reaction at the site of injection [19].

While there have been many reports about the use of CFA in different species, most of them are reported on small animals such as rodents and rabbits [11, 18], which have very different skin morphology from human skin. Here, we describe our experience of the use of CFA in a clinically relevant porcine model of induced skin inflammation.

In this study, we demonstrate that the degree of inflammation and pain affecting the well-being of the animal depends more on the depth of injection than the dose and volume of CFA injected. We used weight loss, diminished food consumption, body temperature, pain, and general condition as indicators of distress in our animals. We injected large amounts of CFA distributed into 4 to 8 sites (each size receiving 0.5ml of 1mg/mL killed mycobacteria), which is two to four times the maximum concentration currently recommended to minimize severe adverse inflammatory reactions [10, 17, 20]. Using these doses, we did not observe any compromise in the welfare of the animals, but we were able to clearly assess the degree of inflammation depending on the route of injection.

Given that weight gain is considered an important indicator of animal well-being, the consistent increase between 10 and 20% observed in the initial weight of all animals over the 44-day study clearly suggests that well-being was not compromised (Table 1), regardless of CFA injection route.

Pain is inferred from scratching behaviors, reduced motor activity of the animal, weight loss, vocalization when the affected site is touched, and a reduction in these behaviors after the administration of opioids. Despite the formation of large granulomas in the deep SC tissue in all SC injected animals, no alteration in behavior, weight loss, body temperature, appetite, pain, or distress were observed. However, this was not the case when animals were injected more superficially (epidermis or superficial dermis) where focal necrosis and ulceration of the skin were observed. These animals showed pain and distress, and although no weight loss was observed, they required pain management and wound care of their skin lesions.

CFA is an oil-based component that when injected diffuses into the surrounding tissue causing an inflammatory reaction. Blood vessels in this respective layer aid in the transport of proinflammatory agents to the circulation. However, in the case of epidermis, a lack of blood supply causes the confinement of CFA and the inability to transport proinflammatory agents, thus a more severe necrosis is observed.

Previous reports have assumed that the development of granulomatous lesions following a SC injection of CFA is painful and distressful for the animals, even though there is no clinical evidence to support this theory [21]. These assumptions are responsible for the strict regulations that are currently imposed on the use of CFA. While we acknowledge, there is a severe inflammatory reaction when CFA is injected superficially, as demonstrated by our (n=2) animals injected intraepidermally or into the superficial dermis, the reaction is not the same, and animals do not show signs of pain and distress when injections are performed deeper.

We found that SC thickness could vary significantly among pigs of similar age, likely due to body score. Pig #7 weighed less than the other pigs, which may have contributed to the unusually thin SC layer in this animal and caused the inadvertent intramuscular CFA injection.

Considering the variations in the pig's skin thickness it would be very helpful to avoid delivery of CFA in an undesired location and therefore avoid compromising the welfare of the animal. Using real-time visualization of needle placement for adequate injection with ultrasound [22] could improve accuracy and confirm the desire delivery location of CFA. Careful selection of the route of injection is therefore crucial to refine immunization protocols and to avoid the potential side effects that could hinder the animal's well-being. When this is achieved, we have demonstrated that animals do not experience pain or distress and their well-being is not compromised. In addition, we have demonstrated that CFA is an adequate model to study cutaneous inflammation in large animals. The inflammatory reaction can be adequately assessed not only by histology, but noninvasively by simply measuring the induration of the lesions which can be accurately done when animals are injected subcutaneously [23]; or by the assessment of oxygen consumption in the lesions as we have previously reported [24]. Thus, this study provides evidence of the safe use of CFA in a humane manner when properly administered into the SC space.

## Figures and Tables

**Table 1:** Table describing the characteristics and demographics of the animals and interventions used.

**Animal**	**Initial weight (kg)**	**Final weight ** **(kg)**	**Percentage of weight change since CFA**	**Amount of CFA injected**	**Route of injection /needle size**	**Pain Medication**	**Appetite Lost**
Pig #1	52.4	63.4	21%	4mL	Intraepidermal/25g	Fentanyl patch	No

Pig #2	48.6	53	9.1%	4mL	Intradermal/25g	Fentanyl patch	No

Pig #3	47	52.2	11.1%	4mL	SC diff depths/25g and 27g w/wo 7mm block/4mL	None	No

Pig #4	46.7	57.4	22.9%	4mL	Subcutaneous/25g	None	No

Pig #5	47.3	44	-0.7%	4mL	Subcutaneous/25g	None	No

Pig #6	41	47	14.6%	4mL	Subcutaneous/25g	None	No

Pig #7	37	45.1	21.9%	4mL	Intramuscular/25g	None	No

## Data Availability

The authors declare that all relevant data supporting the findings of this study are available within the article information files.

## Abbreviations

CFA: Complete Freund's Adjuvant.
